# Prospective investigation of intravenous patient-controlled analgesia with hydromorphone or sufentanil: impact on mood, opioid adverse effects, and recovery

**DOI:** 10.1186/s12871-018-0500-1

**Published:** 2018-04-10

**Authors:** Yanqing Yang, Jianping Wu, Huiling Li, Sujuan Ye, Xiaoying Xu, Ling Cheng, Lina Zhu, Zhiyou Peng, Zhiying Feng

**Affiliations:** 1 Department of Anesthesiology & Pain Medicine, The First Affiliated Hospital, Zhejiang University School of Medicine, 79 Qing Chun Road, Hangzhou, Zhejiang 310003 People’s Republic of China; 2 Department of Anesthesiology, Taizhou Hospital, Linhai, Zhejiang People’s Republic of China; 3 Department of Anesthesiology, The Second Affiliated Hospital of Jiaxing College, Jiaxing, Zhejiang People’s Republic of China

**Keywords:** Hydromorphone, Sufentanil, Intravenous patient-controlled analgesia, Mood, Side effects, Radical surgery for colorectal cancer

## Abstract

**Background:**

Radical surgery for colorectal cancer, associated with moderate to severe postoperative pain, needs multimodal analgesia with opioid for analgesia. Despite considerable advancements, the psychological implications and other side effects with opioid remain substantially unresolved. This study aimed to investigate the impact on mood, side effects relative to opioid, and recovery of the patients with hydromorphone or sufentanil intravenous patient-controlled analgesia (IV-PCA) in a multimodal perioperative analgesia regimen undergoing radical surgery for colorectal cancer.

**Methods:**

Eighty patients undergoing elective laparoscopic or open radical surgery for colorectal cancer under general anesthesia were randomized to receive postoperative IV-PCA with either sufentanil (group S) or hydromorphone (group H). All patients received additionally flurbiprofen axetil 50 mg 30 min before the end of surgery and wound infiltration with 10 ml of 0.75% ropivacaine at the end of surgery. The primary endpoint was mood changes at 48 and 96 h after surgery. The secondary endpoints were the incidence of opioid-related adverse effects, recovery results and patient satisfaction after surgery.

**Results:**

Seventy-two patients completed the study finally. There were no significant differences between the two groups with respect to preoperative parameters, surgical and anesthetic characteristics (*P* > 0.05). No obvious significant differences were observed in VAS score (at rest and during mobilization) and rescue analgesics use (*P* > 0.05). Compared with group S, the anger scores in the group H at 48 h and 96 h after surgery were significantly lower (*P* = 0.012 and 0.005; respectively), but the incidences of pruritus and nausea were higher (*P* = 0.028 and 0.008; respectively). There were no significant differences in the incidences of vomiting, respiratory depression, dizziness, Ramsay score, and hemodynamic changes between the two groups (*P* > 0.05). Moreover, there were no significant differences in the time to gastrointestinal recovery, time to drainage tube removal, time to walk, hospital stay after surgery and patient satisfaction between the two groups (*P* > 0.05).

**Conclusions:**

Under the similar analgesia effect with different opoiods postoperatively, hydromorphone IV-PCA resulted in an improved mood, however, a higher occurrence of pruritus and nausea while compared to sufentanil IV-PCA in a multimodal perioperative analgesia regimen. Both regimens of opioid with IV-PCA may serve as promising candidates for good postoperative pain management, and provide with similar postoperative recovery for the patients undergoing radical surgery for colorectal cancer.

**Trial registration:**

This study was registered with the Chinese Clinical Trial Registry on September 20, 2015 (URL: http://www.chictr.org.cn. Registry number: ChiCTR-IPR-15007112).

## Background

Patients experience moderate to severe pain after colorectal surgery. Effective pain management is a national and global challenge [1, 2]. Inadequately controlled postoperative pain adversely affects patients, resulting in unnecessary physical, psychological, and emotional manifestations [3]. Safe and effective postoperative analgesia not only reduces the postoperative complications but also facilitates rapid recovery and minimizes the emotional suffering including anxiety, fear, depression, disaffection, frustration, and so on [1, 2]. Moreover, a favorable postoperative pain management should provide adequate analgesia with a minimum of side effects [1, 2, 4].

The concept of multimodal analgesia was introduced in the early 1990s and is currently established in clinical practice worldwise. For multimodal analgesia regimen, opioid analgesics are the primary medications for managing pain in hospitalized patients [2, 5, 6]. Intravenous patient-controlled analgesia (IV-PCA) has been demonstrated to be a delivery method that offers significant benefits to patients with postoperative pain after abdomen operation. Opioids IV-PCA are commonly, almost routinely, provided in the immediate postoperative period after numerous types of surgeries. Over the past two to three decades, efforts to improve opioid analgesia have progressed along two following paths: one is to improve opioid analgesia effect, and other is to provide satisfactory pain relief with minimal side-effect burden. Postoperative opioid analgesics have significant adverse effects including nausea, vomiting, excessive sedation, respiratory depression, and so on. Opioids not only severely impair the function of the mu opioid receptors, but also inhibit the release of beta-endorphin resulting in feeling of anhedonia, lead to a relative paucity of dopamine resulting in a depressive mood and lack of “wellness”. Stephan et al. believed that the profound impacts of opioids on mood and wellness are often underscored [7]. Hydromorphone, widely used in the USA and Europe countries, is a semisynthetic mu and delta opioid agonist. It was well demonstrated that hydromorphone has valuable advantages of rapid onset, robust analgesic efficacy, no ceiling effect for analgesia, inactive metabolite, and fewer side effects while compared to morphine [8–10]. Sufentanil, a highly selective mu opioid agonist, provides various advantages such as reliable analgesic efficacy, stable hemodynamics, and few side effects, and has been widely used as an postoperative analgesia in China [11, 12]. Rapp et al. reported that hydromorphone improved the mood of patients while compared to morphine [13]. To our knowledge, more and more studies investigated the different analgesia effect, only a few studies have been investigated on mood changes with different opioids IV-PCA [7, 13, 14]. Therefore, we tested the hypothesis that hydromorphone could improve the postoperative mood for the patients with IV-PCA involved multimodal perioperative analgesia regimen compared to sufentanil. The aim of this prospective comparative investigation was to assess the impact of hydromorphone or sufentanil IV-PCA in a multimodal perioperative analgesic regimen on mood, side effects of opioid and recovery for the patients undergoing radical surgery for colorectal cancer.

## Methods

### Ethics approval and consent to participate

This prospective, double-blind, randomized controlled study was approved by the Ethics Committee of the First Affiliated Hospital of Zhejiang University School of Medicine, Hangzhou, Zhejiang, China (Ethical number: 2015[315]) and registered with the Chinese Clinical Trial Registry (URL: http://www.chictr.org.cn. Registry number: ChiCTR-IPR-15007112). Informed written consent was obtained from all participants before enrollment.

### Criteria for inclusion and exclusion

The study was conducted in the Department of Anesthesiology, the First Affiliated Hospital, Zhejiang University School of Medicine, from September 2015 to February 2016. We enrolled 80 American Society of Anesthesiologists (ASA) physical status I - II patients aged between 18 and 80 years undergoing elective laparoscopic or open radical surgery for colorectal cancer. All were scheduled to receive general anesthesia. Exclusion criteria were as follows: body mass index (BMI) ≤18 or ≥ 30 kg/m^2^, alcohol or drug abuse, history of chronic pain, pre-existing neuralgia, digestive tract ulcer, gastroesophageal reflux or mental diseases, severe renal or hepatic dysfunction, a previous history of delay recovery under general anesthesia, relevant drug allergy, currently taking contraceptives, pregnancy, inability to understand Mandarin, inability to properly describe postoperative pain (e.g. language barrier, neuropsychiatric disorder) or inability to use IV-PCA pump, consumption of opioids within 24 h before surgery, taking monoamine oxidase inhibitor (MAOI) or antidepressant use 15 days before surgery, taking sedative, anti-emetic, or antipruritic during the 24 h preoperatively, anxiety or depressive disorders, transfer to intensive care unit (ICU) after surgery.

### Randomization

The study was a blind randomized controlled trial. The patients were randomly assigned to group S and group H by the random-numbers table. Identical boxes containing either group S or group H were sealed by an independent statistician. One nurse (SJY who did not participate in patient care) opened the box, took out the envelope, prepared the pump, and transfered the pump to the anesthesiologist. Two of the anesthesiologists (YQY, HLL) performed general anesthesia and all intraoperative assessments, and two other investigators (LNZ, LC) assessed and recorded all the postoperative data blinded to the group identity.

### Preoperative preparations and anesthesia protocol

The purpose of this study, IV-PCA instructions and VAS evaluation were explained to the patients 1 day before surgery. Written consent was obtained from participants, and Self-Rating Anxiety Scale (SAS) and Self-Rating Depression Scale (SDS) were evaluated by nurse XYX. The SAS is a 20-item self-report assessment device built to measure anxiety levels, based on scoring in 4 groups of manifestations: cognitive, autonomic, motor and central nervous system symptoms. The SDS is a short self-administered survey to quantify the depressed status of a patient. There are 20 items on the scale that rate the affective, psychological and somatic symptoms associated with depression. Each question is scored on a scale of 1 through 4 (based on these replies: “a little of the time”, “some of the time”, “good part of the time”, “most of the time”). Overall assessment is done by total score. The patients whose standard score of SAS or SDS was ≥50 were excluded from the study. A base line psychometric assessment such as simplified Profile of Mood States (POMS) was performed 1 day before surgery.

All patients received a standardized anesthetic regimen. No additional preoperative medications were administered. Upon arrival in the operating room, ASA standard monitoring, including electrocardiography, invasive blood pressure and pulse oximetry was established. Electrodes were applied to the patient’s forehead for monitoring the bispectral index (BIS) (A-2000 BIS™ monitor; Aspect® Medical Systems, Inc., Natick, MA, USA). After pre-oxygenation for 3 min, anesthesia was induced with midazolam 0.05 mg/kg, propofol 2 mg/kg, and fentanyl 5 μg/kg. Upon confirming the loss of response to verbal orders, 0.6 mg/kg of rocuronium bromide was intravenously administered, and endotracheal intubation was performed after 60 s. Subsequently, mechanical ventilation was initiated to oxygenate the patient with 0.4 FIO_2_ and maintain an end-tidal carbon dioxide of 35–45 mmHg. For anesthesia maintenance, propofol was titrated at 5–8 mg/kg/h with a pump (Double channel micro-infusion pump WZS-50F6; Smiths Medical Instrument Co., Ltd., Zhejiang, China) to keep BIS between 45 and 55, and remifentanil was administrated at 0.05–0.3 μg/kg/min with a pump to keep hemodynamic stable. Boluses of 0.03 mg/kg cisatracurium was intermittently injected to maintain muscle relaxation. Palonosetron hydrochloride 0.25 mg were administered 30 min before the end of surgery. 5 min before the end of surgery, the propofol and remifentanil infusion was stopped. The surgery and local anesthetic infiltration were performed by three surgical teams. Following surgery, the patients were transferred to a post-anesthesia care unit (PACU). Once spontaneous breathing occurred, the neuromuscular blockade was reversed with atropine 0.5 mg and neostigmine 1 mg. Extubation was performed when the patients were conscious and their spontaneous breathing recovered well. The patients were discharged from PACU to the less intensive ward when the modified Aldrete score was minimally 9/10 points.

### Postoperative pain management

A standardized postoperative analgesic regimen was used. Flurbiprofen axetil 50 mg was injected 30 min before the end of surgery intravenously. At the end of surgery, the postoperative wound was infiltrated with 10 ml of 0.75% ropivacaine. The IV-PCA device (Rehn Medtech Ltd., Jiangsu, China) was connected at the end of surgery. For group S, the pump was set to deliver with a loading dose of 3 μg, a bolus dose of 3 μg and a continuous infusion at 1.8 μg/h of sufentanil with a lockout time of 10 min and a maximum dose of 18 μg/h. For group H, the pump was set to deliver with a loading dose of 0.25 mg, a bolus dose of 0.25 mg, a continuous infusion at 0.15 mg/h of hydromorphone with a lockout time of 10 min and a maximum dose of 1.5 mg/h postoperatively. The aim of postoperative analgesia was to control the VAS score below 3 after surgery. If the patient was not satisfied by two or three bolus doses administered continuously by IV-PCA, flurbiprofen axetil 50 mg was administered by surgeon in the ward. In the case of severe side effects, the IV-PCA was stopped. Then the acute pain service (APS) staff would reprogram the pump with decreasing the basal dose and bolus dose by 20–25%. IV-PCA was reconnected after treating and severe side effects subsided.

### Data collection

Patients’ demographic data, the surgery and anesthesia data, including operation mode, surgical procedure, surgeon, operative time, operative incision, estimated intraoperative blood loss, intraoperative fluid replacement, urine volume, anesthetist, anesthesia time (defined as the time spent in the operating room in minutes), extubation time, duration of PACU, the total consumption of anesthetic were recorded.

The VAS score at rest and during mobilization and the consumption of opioid with IV-PCA were measured at the time of discharge from PACU (T_0_), 6 h (T_1_), 12 h (T_2_), 24 h (T_3_), 36 h (T_4_), 48 h (T_5_), 60 h (T_6_), 72 h (T_7_), 84 h (T_8_), and 96 h (T_9_) after surgery. The number of rescue analgesics use was recorded at the following time points: up to T_0_, T_0−_T_1_, T_1−_T_2_, T_2−_T_3_, T_3−_T_4_, T_4−_T_5_, T_5−_T_6_, T_6−_T_7_, T_7−_T_8_, and T_8−_T_9_.

The perioperative mood was evaluated by simplified POMS 1 day before operation and at T_5_ and T_9_ after surgery. The simplified POMS has been used to assess the mood in varied circumstances. The simplified POMS measures self-reported emotional states by rating 40 different adjectives at the time of completion of the test, for instance, “tense,” “depressed,” on a scale of 0 to 4, wherein “0” corresponds to “not at all” (no experience of the feeling) and “4” indicates “extremely” (extreme experience of the feeling). The intermediate ratings include “a little” = 1, “moderately” =2, and “quite a bit” = 3. Furthermore, the simplified POMS yields independent sub-scores for tension, depression, fatigue, vigor, confusion, anger, and self-related emotions.

The side effects including nausea, vomiting, respiratory depression [respiratory rate < 8 per min or pulse oxygen saturation (SpO_2_) < 90%], dizziness, pruritus, the level of sedation, and hemodynamic changes were assessed at T_0_-T_9_. The postoperative sedation was evaluated using Ramsay Sedation Scale (1 = patient is anxious and agitated or restless or both; 2 = patient is cooperative, oriented, and tranquil; 3 = patient responds to commands only; 4 = patient exhibits brisk response to light glabellar tap or loud auditory stimulus; 5 = patient exhibits a sluggish response to light glabellar tap or loud auditory stimulus; 6 = patient shows no response). Hypotension was defined as systolic blood pressure (SBP) 30% lower than baseline or < 90 mmHg.

The time to gastrointestinal recovery, time to drainage tube removal, time to walk, and the duration of hospital stay after surgery were also recorded. The patient satisfaction was assessed at T_3_ and T_5_, and the rating levels were divided into dissatisfied, neutral, satisfied, and very satisfied.

### Endpoint and sample size

The primary endpoint was mood changes at 48 h and 96 h after surgery. The secondary endpoints were the incidence of opioid-related adverse effects, recovery results and patient satisfaction after surgery. A preliminary study was conducted measuring the scores of mood in 10 patients in order to estimate the size of the group essential for the main study. Compared with group S, the score of anger at 48 h after surgery in the group H showed an improvement of 1.51 with a standard deviation (SD) 2.17. Subsequently, the required number of subjects per group was determined as 33 when the α value (level of significance) was 0.05 (two-sided), and the power 1-β was 0.8. We further added an excess of 20% to the sample size in order to compensate for subject attrition, yielding a final group size of 80 patients.

### Statistical analysis

The data were analyzed using SPSS software version 19.0. The Shapiro-Wilk test was used to assess the normal distribution. Parametric data were expressed as mean ± SD or mean with 95% confidence intervals and nonparametric data as a median and interquartile range. Group averages were compared using the Student’s t-test or Mann–Whitney U test as appropriate. For continuous data, the overall differences were tested by one-way analysis of variance (ANOVA) followed by a post hoc test with least significant difference (LSD) t-test or Kruskal–Wallis test followed by a Mann–Whitney U test when appropriate. The categorical data were assessed by Pearson’s chi-square test or Fisher’s exact test. The psychological evaluations were calculated as changes in reference to the baseline. Statistical significance was indicated by *P* < 0.05.

## Results

**Fig. 1:**
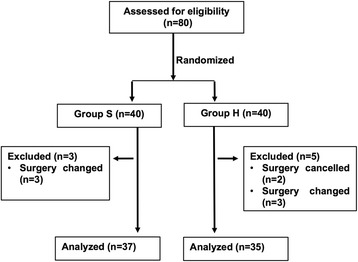
Schematic illustration of the enrolled patients

Eighty patients enrolled in the present study were randomly assigned to the group S (*n* = 40) or group H (*n* = 40). A total of 8 patients were excluded from the final analysis due to surgery cancelled (2 patients), and surgery changed (6 patients). Finally, 72 patients completed the study: 37 in group S and 35 in group H (Fig 1).

### Preoperative parameters, and surgical and anesthetic characteristics

The patients’ demographic and historical variables were similar between the two groups including age, gender, weight, height, education level, ASA status, previous history of surgery, previous history of PCA, diabetes, hypertension, smoking, and motion sickness (*P* > 0.05; Table 1). No significant differences were seen between groups in the terms of operation mode, surgical procedure, surgeon, operative time, operative incision, intraoperative blood loss, intraoperative fluid replacement, and urine volume (*P* > 0.05; Table 2). Moreover, any significant differences were not found with respect to anesthetist, anesthesia time, extubation time, duration of PACU, the total consumption of propofol, fentanyl and remifentanil (*P* > 0.05; Table 3).

**Table 1:** Patients’ demographic and historical variables in both groups

	Group S (*n* = 37)	Group H (*n* = 35)	*P*
Age (years)	53.76 ± 9.73	53.63 ± 12.90	0.962
Gender (Male/female) (n)	25/12	20/15	0.361
Weight (kg)	63.64 ± 10.78	59.26 ± 12.42	0.114
Height (cm)	165.65 ± 8.24	164.17 ± 8.24	0.450
Education level			
Primary / junior /senior/ university (n)	5/22/3/7	5/19/6/5	0.683
ASA status I/II(n)	19/18	17/18	0.814
Previous history of surgery (n)	17	10	0.128
Previous history of PCA (n)	5	0	0.073
Diabetes (n)	2	1	1.000
Hypertension (n)	13	6	0.083
Smoking (n)	11	11	0.875
Motion sickness (n)	2	1	1.000

Values are expressed as mean ± SD or number of patients

*ASA* American Society of Anesthesiologists

**Table 2:** Surgery characteristics

	Group S (*n* = 37)	Group H (*n* = 35)	*P*
Surgical procedure			
Colon carcinoma/rectal cancer (n)	17/20	19/16	0.479
Operation mode			
Laparoscopic/hand-assisted laparoscopic / open (n)	11/8/18	6/12/17	0.325
Surgeon			
Group A/Group B/Group C (n)	9/15/13	10/11/14	0.723
Operative time (min)	178.97 ± 76.35	153.09 ± 58.66	0.110
Operative incision (cm)	6 (5–15)	10 (5–15)	0.272
Intraoperative blood loss (ml)	100 (50–175)	100 (50–100)	0.307
Intraoperative fluid replacement			
Crystalloids (ml)	1689.46 ± 561.42	1502.86 ± 414.43	0.115
Colloids (ml)	500 (100–500)	500 (100–500)	0.612
Urine volume (ml)	150 (125–250)	150 (100–250)	0.900

Values are expressed as mean ± SD, median and interquartile range or number of patients

**Table 3:** Anesthesia characteristics

	Group S (*n* = 37)	Group H (*n* = 35)	*P*
Anesthetist			
Group A/Group B (n)	16/21	18/17	0.487
Anesthesia time (min)	212.95 ± 91.57	181.51 ± 63.88	0.095
Extubation time (min)	31.16 ± 27.71	39.20 ± 18.67	0.717
Duration of PACU (min)	87.11 ± 32.45	90.77 ± 27.00	0.605
Total consumption of propofol (mg)	1053.87 ± 448.71	932.57 ± 352.89	0.208
Total consumption of fentanyl (mg)	0.5 (0.4–0.5)	0.5 (0.4–0.5)	0.922
Total consumption of remifentanil (mg)	2.57 ± 1.35	2.23 ± 0.89	0.220

Values are expressed as mean ± SD or median and interquartile range or number of patients

### Postoperative pain control

**Fig. 2:**
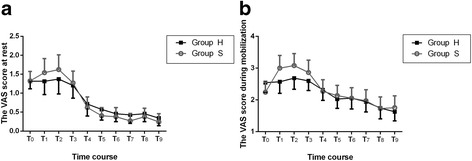
Postoperative VAS score. **a** The VAS score at rest in the two groups of patients during postoperative period. **b** The VAS score during mobilization in the two groups of patients during postoperative period. Values are expressed as mean with 95% confidence intervals. T_0_: discharge from PACU; T_1_: 6 h after surgery; T_2_: 12 h after surgery; T_3_: 24 h after surgery; T_4_: 36 h after surgery; T_5_: 48 h after surgery; T_6_: 60 h after surgery; T_7_: 72 h after surgery; T_8_: 84 h after surgery; T_9_: 96 h after surgery. VAS = visual analogue scale

There was no any significantly difference in VAS score at rest and during mobilization from T_0_ to T_9_ between the two groups (*P* > 0.05, Fig 2). Cumulative consumption of sufentanil with IV-PCA from T_0_ to T_9_ was 5.49 ± 3.14 μg, 17.96 ± 4.79 μg, 29.09 ± 5.36 μg, 51.21 ± 11.21 μg, 70.89 ± 15.79 μg, 91.13 ± 16.72 μg, 110.59 ± 15.25 μg, 127.92 ± 17.40 μg, 138.29 ± 24.22 μg and 148.35 ± 28.31μg, respectively. Cumulative consumption of hydromorphine with IV-PCA from T_0_ to T_9_ was 0.52 ± 0.21 mg, 1.51 ± 0.57 mg, 2.38 ± 0.78 mg, 4.14 ± 1.34 mg, 6.02 ± 2.04 mg, 7.19 ± 1.96 mg, 8.89 ± 2.40 mg, 10.44 ± 2.66 mg, 11.53 ± 2.97 mg and 12.33 ± 3.19 mg, respectively. The number of rescue analgesics use was not statistically significant between the two groups (*P* > 0.05; Table 4).

**Table 4:** The numbers of flurbiprofen axetil for rescue analgesia for two groups

	Group S (*n* = 37)	Group H (*n* = 35)	*P*
up to T_0_	13	8	0.252
T_0−_T_1_	13	14	0.670
T_1−_T_2_	12	14	0.504
T_2−_T_3_	14	14	0.851
T_3−_T_4_	14	14	0.851
T_4−_T_5_	14	16	0.498
T_5−_T_6_	15	16	0.658
T_6−_T_7_	14	16	0.498
T_7−_T_8_	14	16	0.498
T_8−_T_9_	15	16	0.658

Values are expressed as number of patients. T_0_: discharge from PACU; T_1_: 6 h after surgery; T_2_: 12 h after surgery; T_3_: 24 h after surgery; T_4_: 36 h after surgery; T_5_: 48 h after surgery; T_6_: 60 h after surgery; T_7_: 72 h after surgery; T_8_: 84 h after surgery; T_9_: 96 h after surgery

### Mood changes

The primary endpoint was mood changes at 48 h (T_5_) and 96 h (T_9_) after surgery. Compared with the preoperative measures, the scores of fatigue at T_5_ and T_9_ in both groups were higher (group S: *P* = 0.000 and 0.000, respectively; group H: *P* = 0.002 and 0.000, respectively) while the scores of vigor and self-related emotions at T_5_ and T_9_ in both groups were lower (group S: *P* = 0.002 and 0.000, respectively; group H: *P* = 0.007 and 0.000, respectively). And the scores of anger at T_5_ and T_9_ in group S were higher (*P* = 0.001 and 0.000, respectively), whereas the scores of confusion at T_5_ and T_9_ in group H were lower (*P* = 0.003 and 0.003, respectively). But no significant differences were seen in both groups in the terms of tension and depression at T_5_ and T_9_ while compared with the preoperative measures (*P* > 0.05). Compared with group S, the scores of anger at T_5_ and T_9_ in group H were lower (*P* = 0.012 and 0.005, respectively), but the scores of confusion, depression, fatigue, tension, vigor, and self-related emotions at T_5_ and T_9_ were not statistically significant between the two groups (*P* > 0.05; Table 5).

**Table 5:** Profile of Mood States (POMS): changes from preoperative baseline to postoperative 48 h (T_5_) and 96 h (T_9_)

	Group S		Group H	
	T_5_ (*n* = 37)	T_9_ (*n* = 37)	T_5_ (*n* = 35)	T_9_ (*n* = 35)
Anger	1.00 (0.00–2.50)^*^	0.00 (0.00–1.50)^*^	0.00 (−1.00–0.00)^#^	0.00 (−1.00–1.00)^#^
Confusion	0.00 (−2.00–1.00)	0.00 (−1.50–1.00)	−1.00 (−2.00–1.00)^*^	−1.00 (−3.00–0.00)^*^
Depression	0.00 (0.00–2.00)	0.00 (−1.00–2.00)	0.00 (−1.00–1.00)	0.00 (−2.00–2.00)
Fatigue	2.00 (0.00–5.00)^*^	2.00 (0.00–4.00)^*^	1.00 (0.00–3.00)^*^	1.00 (0.00–4.00)^*^
Tension	0.00 (−1.00–2.50)	0.00 (−1.00–1.50)	0.00 (−1.00–1.00)	0.00 (−3.00–2.00)
Vigor	−2.00 (−6.00–0.50)^*^	−3.00 (−6.50–0.50)^*^	−2.00 (−7.00–1.00)^*^	−3.00 (−8.00–0.00)^*^
Self-related emotions	−2.00 (−4.00–0.00)^*^	−2.00 (−5.00–−0.50)^*^	−1.00 (−4.00–1.00)^*^	−2.00 (−5.00–0.00)^*^

Values are expressed as median and interquartile range. T_5_: 48 h after surgery; T_9_: 96 h after surgery

^*^P < 0.05 compared with the preoperative measures; ^#^*P* < 0.05 compared with group S

### Opioid-related adverse effects

A statistically significant difference did not occur between the groups in terms of nausea at T_0_, T_2_–_9_ after surgery, whereas group H showed a significantly higher incidence of nausea than group S during the period from PACU discharge to postoperative 6 h (*P* = 0.008; Table 6). However, no any significant difference was observed with respect to vomiting between two groups (*P* > 0.05; Table 6).

**Table 6:** The number and the incidence rate (%) of postoperative nausea and vomiting in the two groups

	Group S (*n* = 37)		Group H (*n* = 35)	
	Nausea	Vomiting	Nausea	Vomiting
up to T_0_	0 (0%)	0 (0%)	1 (2.9%)	0 (0%)
T_0−_T_1_	2 (5.4%)	1 (2.7%)	10 (28.6%) ^#^	4 (11.4%)
T_1−_T_2_	8 (21.6%)	5 (13.5%)	12 (34.3%)	5 (14.3%)
T_2−_T_3_	5 (13.5%)	3 (8.1%)	8 (22.9%)	3 (8.6%)
T_3−_T_4_	5 (13.5%)	1 (2.7%)	9 (25.7%)	4 (11.4%)
T_4−_T_5_	3 (8.1%)	1 (2.7%)	7 (20.0%)	2 (5.7%)
T_5−_T_6_	3 (8.1%)	2 (5.4%)	8 (22.9%)	3 (8.6%)
T_6−_T_7_	3 (8.1%)	2 (5.4%)	8 (22.9%)	3 (8.6%)
T_7−_T_8_	3 (8.1%)	1 (2.7%)	3 (8.6%)	2 (5.7%)
T_8−_T_9_	1 (2.7%)	1 (2.7%)	2 (5.7%)	3 (8.6%)

Values are expressed as number of patients (%). T_0_: discharge from PACU; T_1_: 6 h after surgery; T_2_: 12 h after surgery; T_3_: 24 h after surgery; T_4_: 36 h after surgery; T_5_: 48 h after surgery; T_6_: 60 h after surgery; T_7_: 72 h after surgery; T_8_: 84 h after surgery; T_9_: 96 h after surgery

^#^*P* < 0.05 compared with group S

**Fig. 3:**
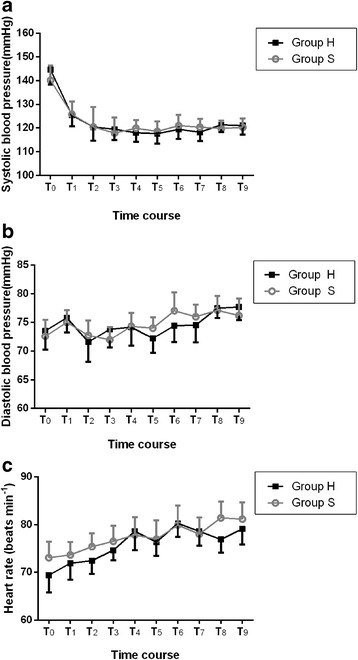
Postoperative hemodynamic changes. **a** Postoperative changes in systolic blood pressure. **b** Postoperative changes in diastolic blood pressure. **c** Postoperative changes in heart rate. Values are expressed as mean with 95% confidence intervals. T_0_: discharge from PACU; T_1_: 6 h after surgery; T_2_: 12 h after surgery; T_3_: 24 h after surgery; T_4_: 36 h after surgery; T_5_: 48 h after surgery; T_6_: 60 h after surgery; T_7_: 72 h after surgery; T_8_: 84 h after surgery; T_9_: 96 h after surgery

Two patients in group S and one patient in group H showed a Ramsay sedation score of 3 while all the other patients presented Ramsay score of 2. In group H, three patients developed hypotension, two patients experienced dizziness, and six patients showed symptoms of pruritus. Conversely, in group S, three patients developed hypotension, but no one experienced dizziness or pruritus. In both groups, none of the patients showed signs of respiratory depression. Moreover, no any significant differences were observed in terms of sedation, dizziness, respiratory depression, and hypotension between the two groups (*P* > 0.05; Table 7); however, when compared with group S, the incidence of pruritus was higher in group H (*P* = 0.028; Table 7). Moreover, there was no any significant difference in the hemodynamic changes during the postoperative period (*P* > 0.05; Fig 3).

**Table 7:** The number of adverse events postoperatively

	Group S (*n* = 37)	Group H (*n* = 35)	*P*
Ramsay score 1 /2 / ≥3 (n)	0/35/2	0/34/1	1.000
Pruritis (n)	0	6^#^	0.028
Dizziness (n)	0	2	0.233
Respiratory depression (n)	0	0	/
Hypotension (n)	3	3	1.000

Values are expressed as number of patients

^#^*P* < 0.05 compared with group S

### Recovery results and patient satisfaction

There were no any significant differences between the two groups with respect to the time to gastrointestinal recovery, time to drainage tube removal, time to walk, or the hospital duration after surgery (*P* > 0.05; Table 8). And no significant differences were observed between the two groups in patient satisfaction at T_3_ and T_5_ (*P* > 0.05; Table 9).

**Table 8:** Comparison of the postoperative recovery parameters

	Group S (*n* = 37)	Group H (*n* = 35)	*P*
Time to gastrointestinal recovery (h)	62.19 ± 26.14	70.27 ± 18.63	0.137
Time to walk (h)	42 (37–52)	43 (38–51)	0.640
Time to drainage tube removal (h)	168.0 (140.5–207.0)	166.0 (142.0–210.5)	0.507
The hospital duration after surgery(d)	9 (7–13)	9 (7–11)	0.596

Values are expressed as mean ± SD or median and interquartile range

**Table 9:** Patient satisfaction at 24 h (T_3_) and 48 h (T_5_) after surgery in the two groups

	Group S		Group H	
	T_3_ (*n* = 37)	T_5_ (*n* = 37)	T_3_ (*n* = 35)	T_5_ (*n* = 35)
Dissatisfied	0 (0%)	0 (0%)	1 (2.9%)	1 (3.2%)
Neutral	11 (29.7%)	5 (11.1%)	7 (20%)	4 (11.4%)
Satisfied	24 (64.9%)	30 (83.3%)	23 (65.7%)	27 (87.1%)
Very satisfied	2 (5.4%)	2 (5.6%)	4 (11.4%)	3 (9.7%)

Values are expressed as number with percent (%). T_3_: 24 h after surgery; T_5_: 48 h after surgery

## Discussion

To our knowledge, this is the first investigation comparing the impact of hydromorphone and sufentanil IV-PCA under similar analgesia effect on the mood after radical surgery for colorectal cancer. We found that hydromorphone IV-PCA was associated with lower anger score, higher incidence of postoperative pruritus and nausea and comparable postoperative recovery compared to sufentanil IV-PCA.

The ideal opioid would be one that provides rapid pain relief with moderate duration while at the same time producing minimal physiologic or psychologic side effects. Psychological processes directly influence clinical outcomes [15]. Sufentanil and hydromorphone are opioid analgesics currently widely used in clinical anaesthesia and postoperative analgesia [8, 10–12, 16]. Lots of literature paid much attention on their analgesia effect or physiological side effects, but less on their side effect on the psychologic issue [7, 13, 14]. Stephan et al. believed that the profound impacts of opioids on mood and wellness are often underscored [7]. Rapp et al. reported that hydromorphone improved the mood of patients while compared to morphine [13]. Sufentanil has been also widely used for postoperative analgesia currently [11, 12, 16]. The aim of this investigation was to compare the impact of hydromorphone and sufentanil IV-PCA under similar analgesia effect on physiologic or psychologic side effects simultaneously. Our result showed that the scores of anger at 48 h and 96 h after surgery in group H were significantly lower when compared with group S, though there were no significant differences in confusion, depression, fatigue, tension, vigor and self-related emotions between the two groups. To eliminate bias and increase comparability, we tried to keep the external conditions and procedures identical between the two groups. The preoperative anxiety or depression might influence the postoperative pain sensitivity, thus aggravating the patients’ subjective feeling of postoperative pain and resulting in an increased demand of the postoperative analgesic [17]. In order to alleviate the impact of the preoperative anxiety or depression on our study, we excluded patients with anxiety or depression preoperatively. The patients with chronic pain, pre-treatment with analgesics or history of drug abuse were also excluded. In our study, no statistically significant differences were found between the groups with respect to the operation mode, surgical team, hemodynamic changes, consumption of anesthetics, and VAS score postoperatively. The opioids have been implicated in modifying the mood states, modulating the mood reactions [18–24], and exercising mood-enhancing properties such as euphoria [22], thereby reducing mood disturbances [23]. However, exogenous opioids halt production of beta-endorphins as well as down-regulate and impair the function of mu opioid receptors [25–27], indirectly limit the action of dopamine in the central nervous system, leading to a feeling of anhedonias [7]. This also might be attributed to the wide distribution of the opioid receptors in the limbic system and locus coeruleus, which are deemed responsible for reward processing and mood control and mental activity [28]. Moreover, accumulating evidence from animal research reveals that mu, delta and kappa opioid receptors exert highly distinct controls over mood-related processes [29]. Delta opioid receptor agonists and kappa opioid receptor antagonists have promising antidepressant potential. Although the mu opioid receptor agonists present strong analgesic effect and sedation, they also cause side effects such as respiratory depression, constipation, and euphoria. The kappa opioid receptor agonists not only provide analgesia but also suppress addiction, whereas the delta opioid receptor agonists possess a strong analgesic activity as well as anti-anxiety, anti-depression, and organ protection [30, 31]. Hydromorphone exhibits a high affinity to mu opioid and delta opioid receptors, whereas sufentanil is a highly selective mu opioid agonist and possesses a low affinity towards delta opioid receptor. Therefore, the speculated underlying mechanisms of improved mood caused by hydromorphone IV-PCA were associated with pain relief induced by the excited mu opioid receptors and the anti-anxiety and anti-depression caused by excited delta opioid receptors; however, further studies are essential to substantiate these findings.

Postoperative opioid IV-PCA have physiologic adverse effects including nausea, vomiting, pruritus, excessive sedation, respiratory depression, and so on. There were no significant differences in the incidences of vomiting, excessive sedation, respiratory depression and hypotension between the two groups. However, the incidence of nausea during the period from PACU discharge to postoperative 6 h was higher in group H when compared with group S. The incidence of pruritus was higher in group H compared to group S. Evidence shows that PONV is caused by multiple factors, including the anesthetics, operation mode and risk factors of the patients [32]. There were no obvious significant differences in the anesthetics, intraoperative opioid, operation, gender, laparoscopic surgery ratio, and motion sickness between the two groups. The Apfel simplified risk score is based on four predictors: female, history of PONV and/or motion sickness, nonsmoking status, and use of postoperative opioids [33]. Roberts et al. and Shaikh et al. demonstrated that postoperative opioids increased risk for PONV [34, 35]. Therefore, we speculate that the signifcant difference in the incidence of nausea between the two groups may result from the different opioid with IV-PCA. Multiple factors may contribute to opioid induced nausea and vomiting, including activation of opioid receptors in the chemoreceptor trigger zone, vestibular apparatus, and gastrointestinal tract [36]. Coda BA et al. found that there was no significant difference in nausea while compared hydromorphone, morphine and sufentanil IV-PCA in patients with oral mucositis pain following bone marrow transplantation [37]. Pruritus is an unpleasant sensation leading to scratching, which may seriously affect the quality of life. Opioids are one of the best-known medications evoking pruritus. It was well demonstrated that pruritus was one of the most common side effects as being the reason for the change in opioid from morphine to hydromorphone [10]. However, to our knowledge, there was no relative report to compare the incidence of pruritus between hydromorphone and sufentanil. Although there were several differences in physiologic adverse effects between hydromorphone and sufentanil IV-PCA, the recovery parameters including the time to gastrointestinal recovery, time to drainage tube removal, time to walk, the hospital duration after surgery and the patient satisfaction were similar. Opioid-induced pruritus and PONV are unwanted side effects, negatively affecting patient functional outcomes, decreasing patient satisfaction and increasing costs. It is worth further investigation.

There are several limitations in this study. First, we have not recorded the awareness of disease of patients before or after surgery, which would also affect the mood and recovery postoperatively. Second, the patients with anxiety and depression were excluded from the study preoperatively, whether hydromorphone IV-PCA can improve the mood of these patients is yet to be elucidated.

## Conclusions

In conclusions, under the condition with similar analgesia effect with different opoiods postoperatively, hydromorphone IV-PCA resulted in an improved mood, however, a higher occurrence of pruritus and nausea while compared to sufentanil IV-PCA in a multimodal perioperative analgesia regimen. Both regimens of opioid for IV-PCA may serve as promising candidates for the management of postoperative analgesia with similar postoperative recovery for the patients undergoing radical surgery for colorectal cancer.

## Abbreviations

ANOVA: One-way analysis of variance
APS: The acute pain service
ASA: American Society of Anesthesiologists
BIS: Bispectral index
BMI: Body Mass Index
DBP: Diastolic blood pressure
ICU: Intensive care unit
IV-PCA: Intravenous patient-controlled analgesia
LSD: Least significant difference
MAOI: Monoamine oxidase inhibitor
PACU: Post-anesthesia care unit
PCA: Patient-controlled analgesia
POMS: Profile of Mood States
PONV: Postoperative nausea and vomiting
SAS: Self-Rating Anxiety Scale
SBP: Systolic blood pressure
SD: Standard deviation
SDS: Self-Rating Depression Scale
SpO_2_: Pulse oxygen saturation
VAS: Visual analogue scale
